# 临床决策支持系统在血液系统疾病中的应用及挑战

**DOI:** 10.3760/cma.j.cn121090-20251012-00460

**Published:** 2026-05

**Authors:** 傲 贾, 丽红 刘, 素萍 秦, 翔宇 赵

**Affiliations:** 1 北京大学人民医院，北京 100032 Peking University People's Hospital, Beijing 100032, China; 2 北京市昌平区医院，北京 102200 Beijing Changping Hospital, Beijing 102200, China

## Abstract

血液系统疾病，尤其是血液肿瘤，具有高度异质性，临床表现多样且生物学特征复杂。临床决策支持系统（clinical decision support system, CDSS）是整合临床数据、医学专业知识与人工智能（artificial intelligence, AI）的辅助诊疗系统，有望提升血液系统疾病诊疗规范性、精准度与效率，并改善预后。本综述系统梳理了CDSS在血液系统疾病中的应用现状，探讨其核心价值，分析当下面临的挑战，并对未来发展进行展望。CDSS在血液肿瘤、出凝血疾病等血液系统疾病的规范诊疗、精准诊治、早期预防、提升效率等方面具有潜力，但面临数据处理、知识库构建与维护、模型性能优化、系统集成、评价标准缺乏等多方面挑战。未来，CDSS有望融合多中心、多模态数据实现精准化、个体化、全程化血液系统疾病管理。

## Abstract (en)

Hematological diseases, particularly hematological neoplasms, are highly heterogeneous, characterized by diverse clinical manifestations and complex biological features. Clinical Decision Support System（CDSS）is an auxiliary diagnosis and treatment system that integrates clinical data, medical expertise, and artificial intelligence（AI）, which is expected to significantly improve the standardization, accuracy, and efficiency of diagnosis and treatment for hematological diseases and thereby improve prognosis. This review aimed to systematically summarize the application status of CDSS in hematological diseases, discuss its core value, analyze the current challenges, and outline future development. CDSS has potential in standardized diagnosis and treatment, accurate diagnosis and treatment, early prevention, and efficiency improvement of hematological diseases, including hematological neoplasms and bleeding and coagulation disorders. However, it faces many challenges, such as data processing, knowledge base construction and maintenance, model performance optimization, system integration, and a lack of evaluation criteria. In the future, CDSS is expected to integrate multicenter and multimodal data to enable precise, individualized, and whole-process hematological disease management.

随着人工智能（artificial intelligence, AI）的高速发展，临床决策支持系统（clinical decision support system, CDSS）作为集医学专业知识、患者数据与AI于一体的重要辅助诊疗工具，正深刻变革着血液系统疾病的诊疗范式。血液系统疾病，尤其是血液肿瘤，具有高度异质性，诊疗复杂，其传统诊疗模式面临巨大挑战。CDSS能够在疾病诊断、危险分层、治疗决策及预后评估等多个关键环节提供精准化、个体化建议，从而有效提升诊疗的规范性、精准度和效率，并改善预后。本综述旨在系统梳理CDSS在血液系统疾病中的应用现状，探讨其核心价值，分析当前面临的挑战，并对未来发展进行展望，为推动其深入应用提供参考。

一、CDSS的概念、发展与功能架构

1. 定义：CDSS是一种利用临床数据、循证医学知识和AI等来辅助医务人员做出更优临床决策，以提升医疗保健水平的信息系统[1]–[3]。

2. 发展与演变：CDSS自问世以来经历了显著的演进，从早期基于规则的专家系统发展到如今的AI驱动系统，其发展与演变的主要历程及特点见表1。

**表1:** CDSS发展与演变历程

技术阶段	核心技术	运行原理	优势	局限性	举例
基于规则的专家系统	预定义的“如果-那么（if-then）”逻辑规则、医学知识库	医学专家将知识编码为明确的逻辑规则；系统将患者数据与规则条件进行匹配，若匹配成功则触发对应的建议或警报	逻辑透明，结果可解释性强；实现简单	知识库维护成本高；难以处理复杂、不确定性问题；无法从数据中学习进化	用于儿科电话分诊的“PED-IA”[4]
基于ML的系统	逻辑回归、支持向量机、随机森林等	从大量已标注的历史临床数据中自动学习诊断模式或预测模型。系统应用学到的模型对新患者数据进行分析、诊断或预测	能从数据中学习模式，处理能力优于规则系统	依赖高质量的结构化数据；特征工程复杂	一款辅助新生儿脓毒症治疗中抗生素用药决策的CDSS[5]
基于DL的系统	卷积神经网络、循环神经网络等	通过多层神经网络结构自动提取数据的层次化特征	能自动提取特征，处理非结构化数据（图像、文本）能力强大	“黑箱”效应显著，可解释性差；计算资源需求高	用于鉴别儿童多系统炎症综合征和地方性斑疹伤寒的“AI-MET”[6]
基于LLM的系统	LLM、知识图谱、检索增强生成等	基于在海量文本和数据上预训练的基础模型，可深入理解临床语言，进行推理并生成符合逻辑的文本内容	交互自然，能处理极其复杂的非结构化数据；具备强大的推理和生成能力	“幻觉”风险；数据隐私与安全要求高；模型训练与部署成本高；对伦理和法律框架提出新挑战	用于识别用药错误的CDSS[7]

**注** CDSS：临床决策支持系统；ML：机器学习；DL：深度学习；LLM：大语言模型

CDSS的出现可以追溯到20世纪70年代，当时研究人员开始使用AI技术开发专家系统。此类专家系统主要基于规则，依赖医学专家以“如果-那么（if-then）”的逻辑规则形式进行知识编码。随着20世纪90年代至21世纪初电子健康档案（electronic health record, EHR）的普及，CDSS与EHR的集成使CDSS能够充分获取患者数据，从而根据患者的个体信息提供个体化建议。20世纪90年代后期，CDSS纳入了循证医学指南和临床实践建议，帮助临床医师根据最新研究证据做出决策，进一步推动了CDSS的功能完善。

21世纪初至今，CDSS的核心架构与功能随着AI的飞速发展而持续演进。从机器学习（machine learning, ML），到深度学习（deep learning, DL），再到大语言模型（large language model, LLM），AI驱动的CDSS通过利用大规模数据集和先进算法，可以提供更为准确且个体化的决策支持。需要强调的是，CDSS的演进并非后一阶段完全取代前一阶段，而是呈现出​​融合共存​​的趋势。例如，一种结合专家知识驱动与ML驱动的混合式AI-CDSS对心力衰竭具有较高的诊断准确性，可用于辅助心力衰竭的诊断，尤其是在缺乏心力衰竭方面专家时[8]。

3. 主要组成部分：

（1）数据库：数据库是CDSS的基础，用于模型的训练、验证与优化，包括各类高质量、标准化的医疗数据，如EHR、检验结果、影像学检查图像及报告、病理学图像及报告、细胞遗传学及分子生物学数据等。

（2）知识库：知识库是CDSS专业性的重要保障，包括但不限于法律法规、国家认可的药品说明书、医疗器械注册证、临床路径、临床诊疗指南、技术操作规范与标准、医学教材、专家共识、专著、文献等。临床知识库应及时更新，更新周期一般不长于半年。

知识图谱（knowledge graph, KG）[9]作为一种结构化、智能化的语义网络，正逐渐形成现代CDSS知识库的重要底层架构。它通过“节点”（代表疾病、药物、症状等医学实体）和“边”（代表实体间的关联），将离散的医学知识进行网状互联，使系统具备了处理复杂医学逻辑和深层语义推理的能力。基于KG的CDSS已应用于高血压用药知识管理和决策支持[10]、胃癌治疗决策支持[11]等领域。

（3）推理引擎：推理引擎是CDSS的核心计算模块，负责将输入的患者个体数据与知识库中的规则或模型进行匹配、运算和逻辑推演，从而生成特定的决策建议。根据底层运算机制的不同，推理引擎主要包括：规则驱动的推理引擎（如“PED-IA”[5]）及AI驱动的推理引擎（如“AI-MET”[7]、COMBINE AF的一项子研究[12]）。

（4）人机交互接口：人机交互接口是CDSS的前端输入与输出模块，是连接后台计算系统与医师终端的“桥梁”，也是CDSS与医院现有信息系统深度集成的关键要素。它不仅负责接收医师输入的临床查询和非结构化补充数据，还将推理引擎生成的建议、提示、警报等信息以可视化、易于理解的格式呈现给医师，是CDSS与医院现有信息系统（如EHR）深度集成、融入日常临床工作流的关键要素。医师的采纳、修改、忽略等反馈信息也通过人机交互接口传输回CDSS，从而优化系统性能。

4. 核心功能：

（1）辅助诊断：CDSS在辅助医学诊断方面具有潜在价值。一项回顾性、纵向观察性研究显示，结合“*BMJ*最佳实践”的CDSS提高了临床医师诊断的准确性，确诊时间和住院天数缩短与CDSS的实施相关[13]。一项随机对照试验中，家庭医学住院医师使用DXplain CDSS可以提升诊断的准确性[14]。

（2）辅助治疗：老年或肿瘤患者的并发症或合并症较多，病情及用药复杂，因药物相互作用等导致的不良事件发生风险较高。CDSS可通过监测、警报等方式，提升治疗的安全性或有效性。研究显示，具备药物警示与提醒功能的CDSS可以减少用药错误[15]。比利时鲁汶大学医院开发了一款用药监测CDSS——“Check of Medication Appropriateness（CMA）”，该系统能够生成精准的用药警报，并得到了较高的医师接受率，可优化药物治疗方案[16]。将CDSS应用于重症监护病房危重症患者的血糖管理，可以将更多患者的血糖水平控制在目标范围内，从而提升血糖管理的质量[17]–[18]。

（3）提高效率：CDSS的实施能够显著提升医院工作效率和成本效益，“TheraDoc”可实时整合数据、自动生成抗菌药敏图谱和结构化临床文档，使原需数天完成的手工任务在不到1 h内完成[19]。一项整群随机对照试验显示，整合到计算机化医嘱录入系统中的CDSS可在不增加诊断错误的情况下，增强基层医疗中实验室检查订购的适当性并减少实验室检查订购量，节约医疗成本[20]。CDSS还可根据语音生成病历文本，节约病历书写时间，提高医疗效率，例如北京大学人民医院发布的“Pai Assistant”。

二、CDSS在血液系统疾病中的应用

1. 血液肿瘤：血液系统肿瘤具有异质性高、病程长、用药复杂等特点，加之相关诊断和治疗技术发展迅猛，​​对医师知识更新提出更高要求​​，增加了其精准诊断和治疗难度。CDSS可促进血液肿瘤精准诊治。瑞典研究团队设计了一款交互式、基于网络的CDSS，可支持会诊准备、多学科讨论和临床决策。该系统改善了卡罗林斯卡大学医院淋巴瘤专业的分子肿瘤委员会会诊中的多模态数据解读[21]。另有研究团队开发出一款基于R语言的开源分析工具包，用于评估急性淋巴细胞白血病（acute lymphoblastic leukemia, ALL）的2年维持治疗阶段中抗代谢药物的滴定；将该工具包整合到CDSS中，可实现对药物滴定实践的实时检查和过程校正，从而提高ALL治疗的安全性和有效性[22]。化疗处方决策支持系统（chemotherapy prescription decision support system, CPDSS）是一种基于规则的CDSS，可通过提供警示信息，提高医师对标准化疗方案的依从性，减少ALL患儿化疗处方错误，从而提高治疗的安全性[23]。在罕见血液肿瘤方面，Blasiak等[24]的研究显示，“CURATE.AI”可动态指导华氏巨球蛋白血症患者使用布鲁顿酪氨酸激酶抑制剂依鲁替尼的治疗，展现出CDSS在辅助罕见血液肿瘤患者个体化精准治疗方面的潜力。

2. 非恶性血液系统疾病：

（1）血栓性疾病预防：我国研究团队采用自然语言处理（natural language processing, NLP）算法驱动的静脉血栓栓塞症（venous thromboembolism, VTE）风险评估CDSS——“DeVTEcare”，其在不同的临床场景中很好地识别出了VTE，尤其是在手术科室、VTE低风险科室和年龄≤65岁的患者中[25]。日本研究团队开发了一款用于VTE风险评分的CDSS，对接受下肢骨科手术患者的回顾性分析显示，该系统得出的总评分与VTE发生率呈显著正相关，表明其可用于术前评估，以辅助判断患者是否需要接受VTE预防[26]。美国一项Meta分析显示，CDSS的使用不仅增加了接受VTE充分预防的外科手术患者的比例，还与VTE事件的减少相关[27]。俄罗斯研究团队基于国内外VTE防治指南开发并应用了一款CDSS，显著降低了医院获得性VTE的发生率[28]。一项随机对照试验显示，一款基于移动应用程序的CDSS——“DECIDE-COAG”，可提高抗凝处方和VTE管理的准确性[29]。

CDSS可促进抗凝药物的精准化、个体化管理，提高抗凝治疗的有效性和安全性。一项整群随机对照试验显示，在基层医疗卫生机构应用一款非瓣膜性心房颤动（房颤）CDSS——“GP-CDSS-2.0”，可提高房颤患者接受适当抗凝治疗的比例，并可帮助房颤患者向合理抗凝治疗过渡[30]。一款基于规则和适应患者个体反应的CDSS被用于房颤消融术中的肝素给药，改善了术中治疗性抗凝状态的维持[31]。门诊房颤CDSS可提高医师开具抗凝处方的指南依从性，并减少严重出血事件发生[32]。一款用于检测抗凝药重复使用的CDSS可降低用药错误的发生频率和持续时间，提高治疗的安全性[33]。加拿大一项前后对照研究表明，在门诊血液透析患者中实施CDSS辅助策略进行抗凝管理是有效的，可能会减少INR检验频率，节约医疗成本[34]。

（2）药源性血小板减少症：在辅助诊断药源性血小板减少症方面，联合多指标警报的CDSS表现优于仅基于实验室结果警报的CDSS。一款基于血小板计数的CDSS可于患者血小板计数下降≥50%时发出警报，以期改善肝素诱导的血小板减少症（heparin-induced thrombocytopenia, HIT）的早期识别，但研究结果表明，该系统因不适当的警报导致了警报疲劳，并且没有改善基于证据的HIT检测[35]。与之形成对比的是，一款联合药物与实验室结果进行警报的CDSS，可有效识别重症监护病房中的药源性血小板减少症[36]。

（3）贫血：贫血患病率高、病因复杂，采用CDSS辅助贫血诊治可改善贫血诊治现状。全球疾病负担研究显示，2021年全球各年龄段贫血患病率约为24.3%（患病例数约19.2亿）[37]。荷兰一项研究表明，内置自动化算法的CDSS可有效帮助基层医疗机构的贫血诊断工作，有助于更全面发现贫血病因[38]。铁缺乏是贫血常见的原因，一款基于规则的CDSS在辅助诊治铁缺乏方面显示出较好的准确性和稳定性[39]。另有研究显示，CDSS可改善成人镰状细胞病患者输血性铁过载的基层医疗管理[40]。

（4）脓毒症：脓毒症被定义为宿主对感染反应失调导致的危及生命的器官功能障碍[41]–[42]，是血液科医师在临床工作中面临的重要挑战。CDSS在辅助脓毒症的诊断与预测方面具有潜在价值。有研究团队开发出一款高效的CDSS，通过ML模型分析全血细胞计数及分类数据，可在重症监护环境中增强脓毒症的快速、准确识别，提高护理和治疗的及时性[43]。另有研究团队开发出一款脓毒症CDSS，可有效识别急诊科可疑脓毒症患者[44]。美国加州大学一项研究通过ML算法，基于生命体征和白细胞计数成功预测高乳酸血症及脓毒症患者的死亡风险[45]，为开发脓毒症CDSS提供了重要依据，有助于优化早期风险分层并指导个体化治疗。

三、CDSS在血液系统疾病中应用的挑战

尽管CDSS在血液系统疾病领域前景广阔，但在某些情况下，CDSS的表现并不理想。在德国3所大学医院的26个科室进行的一项整群随机对照试验显示，欧洲放射学会CDSS——“iGuide”没有减少学术型医院中医师不合理的影像学检查请求的数量[46]。美国TrACER整群随机对照试验发现，基于指南的自动化CDSS对接受骨髓抑制化疗的肿瘤患者初级预防性集落刺激因子使用及发热性中性粒细胞减少率无显著改善[47]。CDSS在构建与应用过程中仍面临一系列挑战，其设计和性能有待优化。

1. 数据整合与质量保障：构建高效CDSS的首要挑战在于数据层面。血液系统疾病诊疗涉及电子病历、血常规及网织红细胞计数、外周血涂片细胞形态学检查、骨髓穿刺液涂片、骨髓活检及细胞化学染色、组织病理学检查、流式细胞术、细胞遗传学及分子生物学检查（如染色体检查及基因诊断）、生化及免疫学检查、出血性疾病检查、溶血性疾病检查、影像学检查等多模态信息。这些数据格式不同、标准各异，整合难度大。四川大学华西医院李为民教授团队基于24 107例患者的真实世界数据集，成功整合了多模态数据，开发出一款可用于肺部感染诊断及预后预测的AI模型[48]。当然，血液系统疾病涉及数据的模态更多、更复杂，因此融合多模态数据是构建血液系统疾病CDSS的重大挑战。还有，医疗数据的质量参差不齐，存在数据缺失、错误等问题，需严格清洗和校正。此外，全程保护患者数据安全与隐私也是一大考验。

2. 知识库构建与维护：构建权威、动态的血液专科知识库面临挑战。血液系统疾病知识，尤其是血液肿瘤知识，更新速度快，临床指南、专家共识及其他循证医学证据不断演进，这要求知识库必须具备高效的更新机制（更新周期一般不超过半年）。KG的构建也面临技术难点，医学术语复杂、多样且存在大量语义关联与嵌套关系，难以精准结构化。

3. AI模型技术难点：平衡模型算法的准确性与可解释性也是一大挑战。基于DL模型可能具备更高预测准确度，但“黑箱”效应显著，内部决策过程不透明，医师难以理解其推理逻辑，从而降低对系统推荐结果的信任度和依从性。

提升模型的泛化能力与公平性也是一大考验。若训练数据源于特定人群或单一医疗机构，模型对不同种族或地域的患者可能存在算法偏见，导致泛化能力下降，甚至加剧医疗不公平。因此，采用多中心、全面的大量高质量数据是提升泛化能力和公平性的关键。联邦学习（federated learning, FL）是一种分布式ML技术，可允许多家医院或研究机构在无需共享敏感原始临床数据的前提下，协同训练AI模型。各参与方仅需在本地使用自身数据训练模型，然后将模型参数加密后上传至中央服务器进行聚合，从而生成泛化能力更强的全局模型。FL既能有效保护患者隐私，又能充分利用多中心数据提升CDSS模型在不同种族或地域患者中的适用性与公平性。丹麦奥尔堡大学医院研究团队开发了基于FL的时间-事件预测模型，并用霍奇金淋巴瘤患者的真实世界数据集进行了验证，在保护个体数据隐私的同时，保持了与基于非分布式方法模型相当的预测性能[49]。

4. 系统集成：CDSS只有顺利融入临床工作流才能发挥作用。CDSS需要与医院现有信息系统深度集成，但医疗机构间信息系统差异大，数据标准、接口不统一，导致实施难度增大。CDSS的交互设计必须​​符合临床诊疗习惯，以非侵入方式在恰当决策点（如医嘱开具时）提供智能提醒，而非生硬弹窗干扰正常诊疗流程。

值得注意的是，CDSS会产生假阳性警报，过多无关紧要或重复的警报会导致警报疲劳，医师可能会选择忽略甚至禁用这些功能。这不但降低医疗效率，而且会降低医师的信任度与依从性。荷兰一项多中心、单日、横断面研究显示，无论是在抗凝药还是其他药物的用药警报方面，CDSS的警报效率普遍较低，且医院药学人员审核警报的时间投入较高[50]。比利时一项研究显示，药物相互作用CDSS会导致大量假阳性警报，增加警报疲劳的风险[51]。优化CDSS设计、改进算法可解决警报疲劳的问题。瑞士一款半自动化的CDSS通过基于情境的算法和临床药师审查，实现了高警报接受率和低警报负担，有效缓解了警报疲劳，获得了医师的高度认可[52]。

5. 效果评估与临床接受：CDSS的效果评估与临床接受度是检验其成败的关键。目前缺乏统一的CDSS评价标准和质量控制体系，难以客观评估其有效性，一定程度上限制了CDSS的落地应用。如何制定科学的评价标准尚待讨论。

医师对CDSS的信任与依从是充分发挥CDSS效能的重要因素。一项关于CDSS管理终末期肾病血液透析患者肾性贫血效能的研究显示，医师对CDSS的依从性是CDSS效能的完全中间因素；CDSS通过医师对其的依从，降低了贫血管理的失败率[53]。因此，应不断优化系统性能，提升CDSS专业性、准确性，并将其更好地整合到临床工作流中，以提高医师对CDSS的信任度与依从性。

四、总结与展望

海量的临床数据、庞大的知识库、高效的AI算法赋予了CDSS在规范诊疗、精准诊治、早期预防、提升效率等方面的应用潜力。在血液系统疾病领域，CDSS的潜在应用价值目前主要集中在辅助血液肿瘤复杂诊疗决策、血栓性疾病预防、脓毒症早期识别与预警等方面。

但CDSS性能尚不成熟，需攻克从数据、知识、模型、集成到评估的多重难关，包括：整合多模态数据，数据治理，保护数据安全与隐私，高效更新知识库，构建高性能KG，平衡模型准确性与可解释性，提升模型泛化能力与公平性，CDSS与现有信息系统深度集成，优化人机交互设计，克服警报疲劳，建立评价标准等；以提高医务人员的信任度与依从性。

未来，CDSS辅助血液系统疾病管理将向精准化、个体化、全程化​​方向进一步发展。CDSS需进一步融合分析多模态数据，从而实现精准诊断、危险分层与防治。借助AI自我学习能力，CDSS有望实现知识库的自动化及时更新，并通过实时收集、持续挖掘真实世界数据，校准模型、优化算法，从而推动个体化治疗。CDSS可监测、收集、整合患者在不同医院的诊疗数据，推动血液系统疾病全程化防治体系的建设。
